# Migration-driven exposome reconfiguration: a predictive, preventive and personalized medicine perspective on cardiometabolic risk

**DOI:** 10.1007/s13167-026-00462-7

**Published:** 2026-07-08

**Authors:** Livia Alvarenga, Ribanna Aparecida Marques Braga, Júlia Galbiati de Souza, Carla Soraya Costa Maia, Denise Mafra, Elizabeth Aparecida Ferraz da Silva Torres, Nágila Raquel Teixeira Damasceno

**Affiliations:** 1 Department of Cardiopneumology, Faculty of Medicine (FMUSP), University of São Paulo, São Paulo, SP Brazil; 2 Heart Institute (InCor), Faculty of Medicine, University of São Paulo, São Paulo, SP Brazil; 3 Graduate Program in Public Health, State University of Ceará (UECE), Fortaleza, CE Brazil; 4 Graduate Program in Nutrition and Health, State University of Ceará (UECE), Fortaleza, CE Brazil; 5 Graduate Program in Nutrition Sciences, Fluminense Federal University (UFF), Niterói, RJ Brazil; 6 Graduate Program in Medical Sciences, Fluminense Federal University (UFF), Niterói, RJ Brazil; 7 Graduate Program in Biological Sciences – Physiology, Federal University of Rio de Janeiro (UFRJ), Rio de Janeiro, RJ Brazil; 8 Department of Nutrition, School of Public Health, University of Sao Paulo, Av. Dr. Arnaldo, 715, Sao Paulo, 01246-904 SP Brazil

**Keywords:** Exposome, Migration, Migrant health; Environmental pollution; Dietary acculturation; Psychosocial stress; Sleep disruption; Climate-related exposures; Social vulnerability; Barriers to healthcare;, Cardiovascular disease; Reshaping individual exposure profiles; Biological responses;, Cardiometabolic risk assessment and effective prevention;, Predictive preventive personalized medicine (PPPM / 3PM ); The paradigm change; Proactive medicine;

## Abstract

Contemporary global migration, driven by socioeconomic instability, armed conflict, climate change, and political factors, has emerged as a major determinant of exposome reconfiguration and cardiometabolic vulnerability. Migrant populations are disproportionately exposed to cumulative environmental, psychosocial, behavioral, and cultural stressors that may contribute to chronic low-grade inflammation, neuroendocrine dysregulation, metabolic dysfunction, and increased cardiovascular disease (CVD) risk across the life course. Within the framework of predictive, preventive, and personalized medicine (PPPM/3 PM), migration should not be viewed solely as a demographic phenomenon, but rather as a dynamic process capable of reshaping individual exposure profiles and biological responses. Environmental pollution, dietary acculturation, psychosocial stress, sleep disruption, climate-related exposures, social vulnerability, and barriers to healthcare interact to modulate the gut–brain axis, microbiota composition, inflammatory pathways, and cardiometabolic outcomes. This narrative review discusses migration as a driver of exposome-mediated cardiovascular risk and explores how exposome science may support risk stratification, early identification of vulnerable populations, and targeted preventive strategies in migrant individuals. Furthermore, we discuss the potential role of biomarkers, digital health technologies, and multi-omics approaches in enabling more personalized and culturally sensitive interventions. By integrating migration, exposome science, and PPPM principles, this review proposes a shift from generalized, reactive healthcare models toward more proactive, mechanism-based, and precision-oriented approaches to cardiometabolic prevention in migrant populations.

## Introduction

International migration has emerged as one of the most significant demographic processes of the 21 st century, with over 281 million people residing outside their country of birth in 2020 [1]. Beyond a demographic phenomenon, migration represents a dynamic process of exposome reconfiguration, characterized by cumulative changes in environmental, psychosocial, behavioral, and cultural exposures across the life course. These transitions often place migrants in situations of increased vulnerability due to socioeconomic disadvantages, limited access to healthcare, occupational risks, and psychosocial stressors associated with acculturation and displacement [2, 3].

It is important to emphasize that migrant populations should not be considered a homogeneous group, as the term “migrants” encompasses highly heterogeneous populations, including economic migrants, refugees and asylum seekers, undocumented migrants, international students, migrants undergoing family reunification, and populations displaced by climate change [4, 5]. These groups differ in terms of the factors driving migration, socioeconomic conditions, legal status, psychosocial stress burden, access to healthcare, occupational exposures, cultural adaptation processes, and environmental exposures [4, 5].

Within the framework of predictive, preventive and personalized medicine (PPPM/3PM) [6, 7], migration-related exposures are particularly relevant because they may contribute to suboptimal health conditions and subclinical dysfunction before overt disease develops [7, 8]. Air pollution, urbanization, dietary transitions, sleep disruption, social isolation, and chronic psychosocial stress are examples of exposures that accumulate over time and interact with biological systems involved in inflammation, neuroendocrine regulation, metabolic dysfunction, and cardiovascular risk [2, 3].

The exposome concept provides a systems-based framework for understanding how cumulative environmental exposures interact with biological responses across the life course to influence disease vulnerability [9]. This perspective shifts the focus from isolated risk factors toward an integrated understanding of disease vulnerability, integrating external exposures with endogenous mechanisms such as oxidative stress, inflammation, microbiota alterations, and immune dysregulation. In this context, the well-known concept that “genetics loads the gun, but the environment pulls the trigger” becomes particularly relevant [10, 11].

In this context, cardiovascular disease (CVD) remains the most common cause of premature mortality and disability worldwide. According to the Global Burden of Disease Study 2019, the number of prevalent CVD cases nearly doubled from 271 million in 1990 to 523 million in 2019, while annual CVD deaths increased from 12.1 million to 18.6 million over the same period. The burden of disease also rose substantially, with disability-adjusted life years (DALYs) and years of life lost increasing globally, and years lived with disability doubling from 17.7 million to 34.4 million. Ischemic heart disease and stroke remain the major contributors to this burden, accounting for 9.14 million and 6.55 million deaths, respectively, in 2019. These trends highlight the urgent need to identify modifiable determinants of cardiovascular risk and implement effective predictive, preventive, and personalized strategies to reduce the growing global burden of CVD [12]. Although much of this burden is explained by modifiable risk factors, the potential for prevention remains a window to be explored [12, 13]. Environmental determinants play a decisive role; according to the Global Burden of Disease, air pollution has become the leading environmental cause of death, surpassing even some of the classic cardiovascular risk factors [14].

However, despite the growing recognition of the exposome in cardiovascular research, little attention has been paid to how migratory processes reshape exposure profiles and, consequently, disease risk. Migrants often experience abrupt dietary changes, varying degrees of urbanization, traffic-related pollution, and increased risks associated with crime, social isolation, and reduced opportunities for physical activity [11, 15, 16]. These overlapping exposures make migrant populations a critical, yet understudied, group in which the interaction between the exposome and cardiovascular outcomes may be particularly evident.

Given these multidimensional exposures and the growing burden of cardiometabolic diseases among migrant populations, there is an urgent need for integrative frameworks capable of identifying vulnerable individuals before the onset of overt disease. From a PPPM perspective, exposome science may support risk stratification, early detection of subclinical dysfunction, and development of targeted and culturally adapted preventive strategies [6, 17].

We hypothesize that migration-related exposome reconfiguration contributes to cumulative biological vulnerability and suboptimal cardiometabolic health through the interaction of psychosocial, environmental, behavioral, and cultural exposures. Identification of exposure patterns and vulnerable migrant profiles may enable earlier intervention, targeted prevention, and personalized strategies within the PPPM framework. Accordingly, this review proposes an exposome-informed PPPM perspective on cardiometabolic vulnerability in migrant populations and discusses the implications of migration-driven exposures for predictive, preventive and personalized healthcare strategies.

## Migration-driven exposome reconfiguration and cardiometabolic vulnerability in the PPPM era

**Fig. 1:**
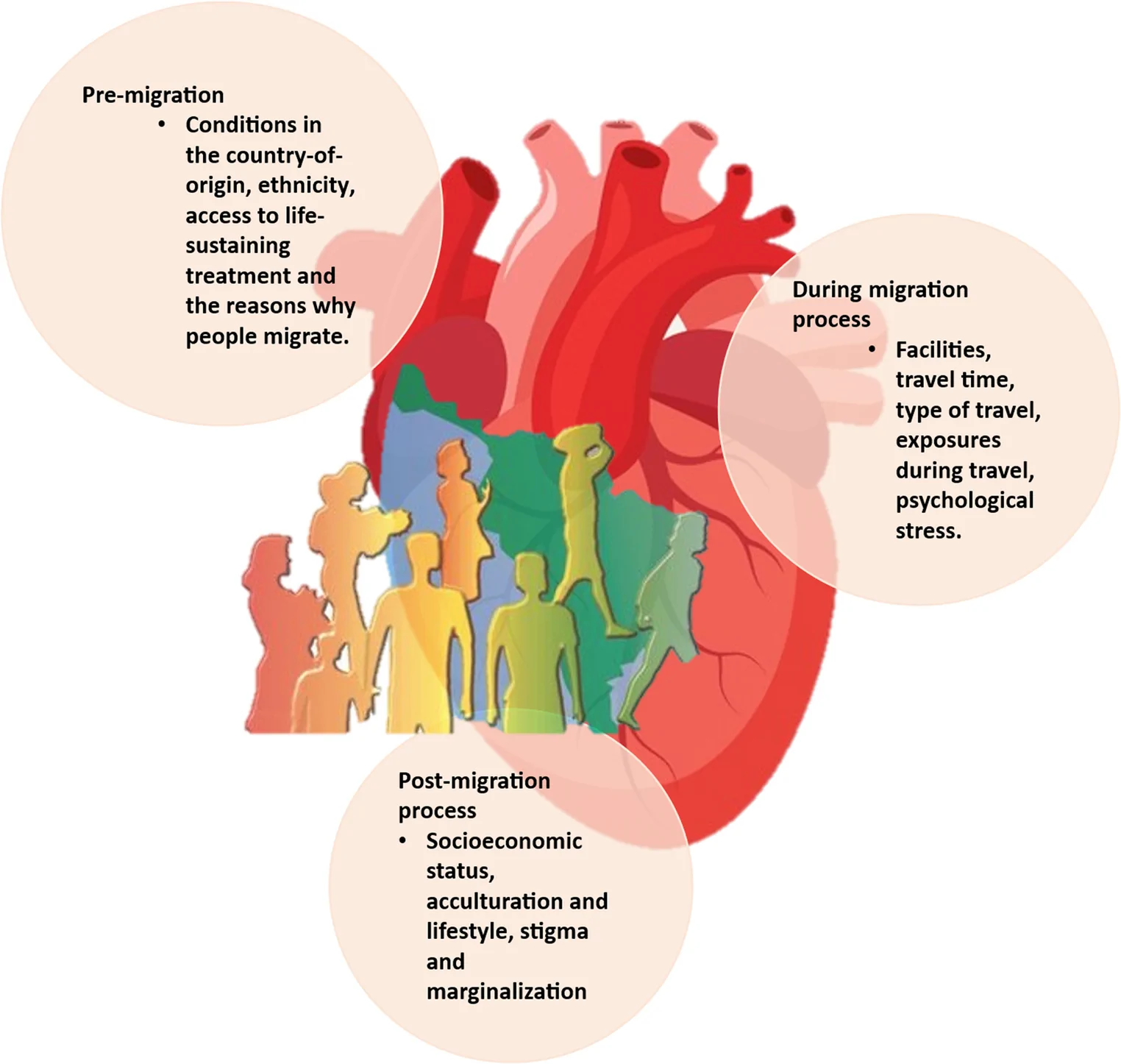
Immigration factors that may influence increased cardiovascular process. In the pre-migration phase, genetic and environmental factors in the country of origin have important relevance for the risk of developing CVD, including individual behavioral risk factors, access to health care, and community determinants of CVD. During the journey, risks related to injury and violence, psychological distress, mental health disorders, and lack of access to care and, especially, life-sustaining medications, are dominant. The migration process, whether voluntary, forced, or illegal, influences the phenotypic characteristics of those who move, in particular, the likelihood that those with pre-existing CVD will move and survive the journey. In the post-migration phase, risk factors for CVD include the conditions in which migrants live in the host countries, barriers to accessing care, and problems adapting to the prevailing culture

According to Odone et al. (2018), three factors influence the burden of CVD: first, social conditions in the country of origin; second, the migration process itself; and third, legal status in the host country (Fig. 1) [2]. Thus, the processes of globalization, urbanization, and migration are part of the complex exposome related to CVD risk; therefore, it is relevant to understand how they influence the burden and nature of CVD [3].

Additionally, individuals may belong to multiple migration categories or transition between migration statuses over time and across different stages of the migration process. Furthermore, individuals may belong to multiple categories or transition between migratory statuses over time, resulting in dynamic and constantly evolving exposure profiles [4].

These subgroups of migrants differ substantially in the factors driving migration, legal status, socioeconomic conditions, work environments, access to healthcare, psychosocial impacts, and cultural adaptation processes. Consequently, they are exposed to distinct combinations of environmental, social, behavioral, and occupational factors that can influence cardiometabolic vulnerability through different biological and psychosocial pathways [4]. Refugees and asylum seekers, for example, are most frequently exposed to displacement, trauma, food insecurity, unstable housing conditions, and chronic psychosocial stress. In contrast, labor migrants are often disproportionately exposed to occupational hazards, shift work, precarious employment, and environmental pollutants. Undocumented migrants may face additional barriers related to legal insecurity, discrimination, social exclusion, and restricted access to health services, while international students may suffer from acculturative stress, social isolation, dietary transitions, and sleep disturbances associated with adapting to a new sociocultural environment [4].

Several population-based studies have explored how migration shapes cardiovascular risk, and the results reveal a complex and often heterogeneous picture. In Ghanaian populations, for example, migration has been associated with accelerated epigenetic aging, with obesity, blood pressure, and insulin levels exerting different impacts depending on the migration context [18]. These findings suggest that migration-related exposures may accelerate biological aging processes before overt cardiometabolic disease becomes clinically apparent. Other evidence suggests that men who moved from Ghana to Europe, particularly to Berlin, had a higher cardiovascular risk than their rural counterparts in Ghana or even Ghanaians in Amsterdam, and that longer residence abroad further amplified this risk [19, 20]. Similar patterns have been described in migrants from South Asia, where the transition to urban environments was followed by a rapid increase in adiposity and, subsequently, in blood pressure and insulin levels, especially among men from disadvantaged backgrounds [21].

However, not all results point in the same direction. As discussed in a study of Portuguese migrants living in Lausanne, Switzerland, they had a cardiovascular risk profile comparable to that of those living in Porto, Portugal, but had better control of hypercholesterolemia [22]. In contrast, Italian migrants in South Australia had higher rates of hypertension, hypercholesterolemia, and smoking than the Australian-born population, while Australian-born Italians had higher obesity, insufficient physical activity, and diabetes prevalence [23].

In addition, refugees represent a particularly vulnerable group, as seen in Afghan refugees, for example, who did not meet the American Heart Association’s standards for optimal cardiovascular health [24]. Mental health has also been highlighted as a critical modifier of cardiovascular outcomes, with links to diabetes, neurological disorders, and CVD documented in migrant groups [25]. In a study of migrants from Sub-Saharan Africa and Southeast Asia in a Greek refugee camp, high levels of post-traumatic stress and depression were strongly associated with metabolic syndrome. Interestingly, better self-perceived physical fitness in this cohort correlated with better psychological well-being and a lower likelihood of metabolic syndrome [26].

Although current evidence remains heterogeneous and predominantly based on cross-sectional designs, accumulating data support the concept that migration-related exposures exert cumulative and biologically relevant effects across multiple stages of the life course [27]. Accumulating data support the idea that migration influences cardiovascular health at multiple stages, such as exposures in the country of origin, during the migration journey, and in the living conditions of host societies. This reinforces the need to situate migration within the broader framework of the exposome to understand how layered environmental and social factors converge to shape cardiovascular disease risk.

From a PPPM perspective, these findings highlight migration as a dynamic determinant of cumulative cardiometabolic vulnerability that may begin long before overt clinical manifestations. In addition, recognition of migration heterogeneity supports the identification of subgroup-specific exposure patterns, improves risk stratification, and facilitates the development of more targeted and culturally sensitive preventive strategies. Identifying migrant-specific exposure profiles, including psychosocial stress, dietary acculturation, urban environmental exposures, sleep disruption, and social deprivation, may support risk stratification and early identification of biologically vulnerable individuals. Furthermore, integrating exposome assessment with biomarkers of inflammation, metabolic dysfunction, neuroendocrine dysregulation, and microbiota alterations may enable more targeted and culturally adapted preventive strategies in vulnerable migrant populations.

## Exposome-informed mechanisms underlying cardiometabolic vulnerability

The exposome concept has emerged as a systems-oriented framework capable of integrating cumulative environmental exposures with endogenous biological responses across the life course, thereby providing new opportunities for understanding cardiovascular vulnerability beyond traditional risk factors [28, 29]. These biological responses may be identified through inflammatory, metabolic, epigenetic, and microbiota-related biomarkers, potentially enabling earlier detection of cardiometabolic vulnerability [30, 31].

The well-established relationship between natural, built, and social risk factors and CVD highlights the potential to modify the environment in favor of preventing conditions that expose individuals to increased chances of developing heart and vascular diseases [32]. Although genetic susceptibility contributes to cardiovascular risk, current evidence indicates that environmental and exposome-related factors play a decisive role in modulating disease expression across genetically susceptible populations [33]. Most chronic cardiometabolic diseases result from complex interactions between genetic susceptibility and cumulative environmental exposures, reinforcing the importance of exposome-informed approaches to disease prevention and risk stratification [34].

**Fig. 2:**
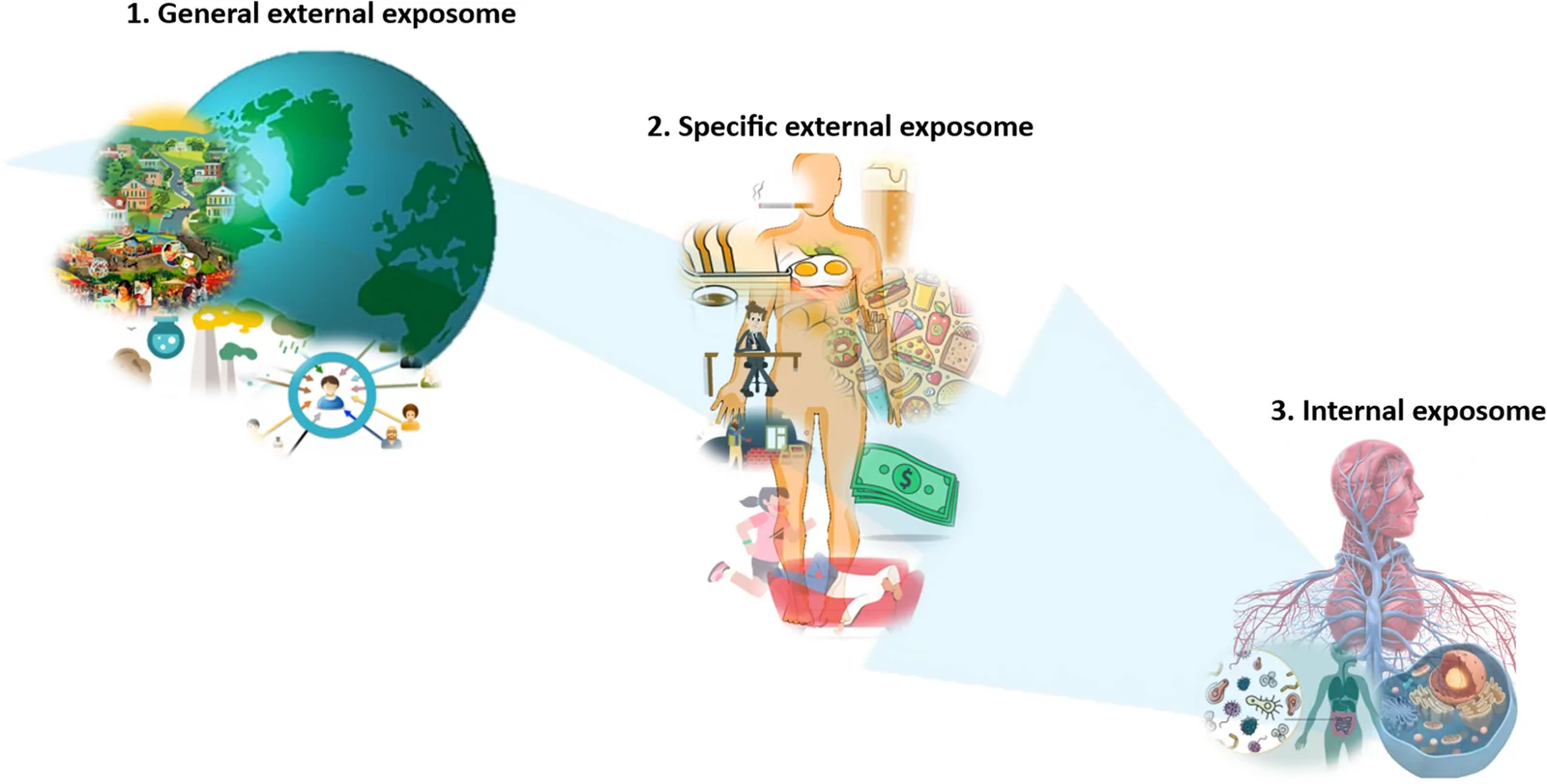
Types of Exposome. (1) General external exposome, encompassing the physical space in which one lives; social environment, being the relationships and social contexts in which groups of people live; physical-chemical environment, chemical or physical agents in our local area; (2) Specific external exposome, being those exposures at the individual level, such as health behaviors, income/financial situation, exposure to pollutants, and lifestyle (smoking, alcohol intake, physical activity), and food environment, encompassing accessibility, availability, and affordability of food in the neighborhood, and (3) Internal exposome, characterized by internal biological processes, including metabolism and microbiome, that can be impacted by external exposures

The exposome includes multidimensional external exposures, including environmental pollution, psychosocial stressors, lifestyle factors, urbanization, and social determinants, as well as internal biological responses involving inflammation, oxidative stress, metabolic alterations, neuroendocrine dysregulation, microbiota composition, and multi-omics interactions (Fig. 2) [35]. These biological alterations may promote endothelial dysfunction, atherosclerosis, vascular inflammation, myocardial remodeling, and metabolic dysregulation, thereby contributing to the development and progression of cardiovascular diseases [36].

Exposome science provides an opportunity to move beyond conventional cardiovascular risk assessment toward more integrative and individualized approaches capable of identifying biologically vulnerable individuals before clinical manifestations occur. Integration of exposome profiling with biomarkers, omics technologies, and digital health tools may support risk stratification, early detection of suboptimal health conditions, and development of targeted preventive interventions in populations exposed to cumulative environmental and psychosocial stressors. Such approaches may contribute to a paradigm shift from reactive cardiovascular care toward earlier, more predictive, preventive, and personalized strategies.

### Social exposome, suboptimal health, and cardiometabolic vulnerability in migrant populations

Migration, both as a process and a condition, fundamentally reshapes the social exposome by exposing individuals to new structural, psychosocial, behavioral, and cultural environments that may modulate cardiometabolic vulnerability in complex and dynamic ways [15]. Social determinants such as housing instability, precarious employment, low income, social deprivation, discrimination, and limited healthcare access disproportionately affect migrant populations and are consistently associated with increased prevalence of hypertension, diabetes, obesity, and metabolic syndrome [37]. Within the framework of predictive, PPPM/3PM, these cumulative exposures are particularly relevant because they may contribute to suboptimal health conditions and biological vulnerability long before overt cardiometabolic disease develops [8].

Although some migrant groups initially present more favorable health indicators than native populations, a phenomenon known as the “healthy migrant effect”, this advantage often diminishes over time, particularly among socioeconomically disadvantaged individuals exposed to chronic psychosocial stressors, discrimination, acculturative stress, and social isolation [38]. From a PPPM perspective, this progressive deterioration may reflect a transition from suboptimal health to clinically manifest disease, driven by cumulative exposome-related burden. Chronic psychosocial stress may induce dysregulation of the hypothalamic–pituitary–adrenal (HPA) axis, increased allostatic load, endothelial dysfunction, oxidative stress, and systemic inflammation, all of which are mechanistically associated with insulin resistance, atherosclerosis, and cardiovascular disease progression [39, 40].

Migration also disrupts social and cultural continuity. Fragmentation of family networks, changes in daily routines, linguistic barriers, loss of cultural identity, and adaptation to unfamiliar social norms may contribute to psychological distress and maladaptive coping mechanisms [39]. These psychosocial exposures interact with biological systems involved in inflammation, neuroendocrine regulation, immune dysfunction, and metabolic homeostasis, reinforcing the concept that migration-related exposures may become biologically embedded over time. Importantly, limited access to culturally adapted healthcare services may further hinder early detection and management of cardiometabolic alterations in migrant populations [40].

The acculturation process itself presents paradoxical effects on cardiovascular health. While social integration may improve access to healthcare and economic opportunities, acculturation is frequently associated with adoption of Westernized dietary patterns, increased consumption of ultra-processed foods, physical inactivity, sleep disturbances, and sedentary behaviors [41, 42]. This phenomenon has been consistently observed among Hispanic populations in the United States, in whom longer residence duration is associated with increased prevalence of obesity, metabolic syndrome, and diabetes, regardless of socioeconomic status [43].

Cultural beliefs, explanatory models of disease, religiosity, and health-related behaviors also play an important role in shaping cardiometabolic risk and healthcare utilization among migrants. Individuals from collectivist cultures may prioritize family or community obligations over preventive healthcare practices or may underrecognize asymptomatic conditions such as hypertension and dyslipidemia [44]. Among South Asian populations, for example, holistic concepts of bodily balance may influence adherence to preventive and therapeutic interventions [41]. These culturally mediated perceptions are highly relevant within PPPM because they may influence health literacy, healthcare-seeking behavior, treatment adherence, and participation in preventive strategies.

The occupational environment represents another critical dimension of the migrant exposome. Migrants are frequently overrepresented in occupations characterized by physical demands, long working hours, shift work, job insecurity, poor sleep quality, irregular eating patterns, and hazardous environmental conditions [45]. A study examining work temporality among Latino workers in the United States demonstrated that conventional employment indicators may underestimate exposure to evening, night, weekend, and irregular work schedules, particularly among non-citizen Latino women, highlighting how nativity and citizenship status shape occupational exposures [46]. Similarly, a study involving healthcare professionals showed that night-shift workers exhibited poorer physical and environmental quality of life, worse psychological health and social relationships, and higher levels of anxiety, stress, and depression compared with day-shift workers [47].

In this context, circadian disruption may alter the rhythmic regulation of cortisol secretion, sympathetic nervous system activity, blood pressure control, glucose metabolism, and inflammatory responses. Persistent misalignment between endogenous biological rhythms and behavioral schedules has been associated with sleep deprivation, autonomic imbalance, insulin resistance, endothelial dysfunction, oxidative stress, and chronic low-grade inflammation [48, 49]. These alterations may accelerate atherosclerotic processes and increase the risk of hypertension, coronary artery disease, myocardial infarction, and stroke [48, 49]. Importantly, undocumented migrants may experience additional barriers related to occupational protection, healthcare access, and social support, thereby amplifying vulnerability and health disparities in this group [50].

Despite the growing recognition of the role of social and cultural determinants in cardiovascular health, research exploring migration-related exposome reconfiguration within a PPPM framework remains limited. Most studies are cross-sectional and concentrated in high-income countries or specific ethnic groups, leaving substantial gaps regarding Latin American, African, and intraregional migrant populations [5, 50].

Furthermore, few interventions have explicitly addressed the cultural and social realities of migrant communities in cardiovascular prevention. Culturally adapted health promotion strategies, community-based participatory research, and the integration of intercultural health mediators remain underutilized. Concepts such as sense of belonging, which predict health behaviors and outcomes, are rarely incorporated into public health frameworks for migrant populations, despite being fundamental to social cohesion and psychological well-being [51].

From a PPPM perspective, migrant populations may present distinct vulnerability profiles characterized by different combinations of psychosocial stress, sleep disruption, social isolation, economic adversity, occupational burden, dietary acculturation, and reduced healthcare accessibility [8].

Accordingly, targeted prevention strategies should move beyond generalized public health recommendations and incorporate culturally adapted, community-based, and personalized approaches. Integration of mental health assessment, eHealth literacy, resilience-promoting interventions, and culturally sensitive healthcare mediation may improve engagement and effectiveness of preventive strategies in migrant populations. Similar PPPM-oriented approaches emphasizing psychosocial profiling, resilience, and tailored interventions have already been proposed in vulnerable populations exposed to chronic psychosocial adversity [52].

### Social vulnerability, healthcare access, and cardiometabolic risk within the PPPM framework

Independent of the motivations underlying migratory movements, migrant populations are frequently exposed to conditions of social vulnerability and cumulative psychosocial burden. Social vulnerability encompasses multidimensional socioeconomic and structural determinants, that may compromise adaptive capacity and resilience in the context of chronic diseases and environmental stressors [53].

Beyond traditional cardiovascular risk factors, social vulnerability has been shown to explain a substantial proportion of geographic variation in cardiovascular morbidity and mortality [48]. Migrants frequently experience barriers to healthcare access in host countries, including language difficulties, limited health literacy, legal insecurity, discrimination, lack of insurance coverage, and unfamiliarity with healthcare systems [1]. Importantly, migrant populations are highly heterogeneous regarding vulnerability profiles, healthcare needs, cultural backgrounds, and disease burden. Forced migrants and refugees, in particular, may present higher levels of psychosocial stress, trauma exposure, food insecurity, sleep disturbances, and chronic inflammation, all of which are associated with increased cardiometabolic and mental health risk [54].

Individuals migrating from low- and middle-income countries may face additional challenges associated with unequal healthcare infrastructure, environmental pollution, and social inequities. Although high-income countries may theoretically provide greater healthcare resources, many healthcare systems remain insufficiently prepared to address the complex psychosocial, cultural, linguistic, and cardiometabolic needs of migrant populations [1]. These findings highlight the importance of identifying vulnerable migrant profiles before the onset of clinically manifest disease. Integration of social determinants, psychosocial assessment, inflammatory and metabolic biomarkers, digital health technologies, and culturally sensitive screening strategies may support earlier identification of individuals at increased cardiometabolic risk [7, 17].

Moreover, this “marginalized” group lives under increased risk, where 92% of pollution-attributable deaths occur [55]. A recent study shows that the negative impact of migratory movement on cardiovascular health exceeds the income variable. According this study, immigrants living in New York city when compared with US-born adults had less access to health insurance (22% versus 12%), self-reported fair or poor health (26% versus 17%) and higher prevalence of hypertension (30% versus 23%) and Type 2 diabetes (T2D) (20% versus 11%) [56].

Thus, Robinson et al. (2025), through a review, demonstrated that it is necessary to understand the opportunities and practical actions to support better access to health for migrants, especially forced migrants [57]. In their work, they elucidated promising strategies to reduce the risk of health problems, both physical and mental. Among these strategies, they highlighted links with formal health services; formation of care networks; proactive engagement, including conducting care in familiar spaces; thoughtful communication; informed providers and improved attitudes; and the right to knowledge, respecting the need of newcomers for information, knowledge, and trust in local systems [57].

It is essential to acknowledge that the vast majority of host countries encounter a significant language barrier, which complicates healthcare and is often intertwined with a range of other policy and service delivery issues. In fact, solutions to bridge this gap are therefore needed, including the use of technologies to overcome this barrier [58]. In this context, digital health technologies, translation tools, telemedicine, and eHealth platforms may represent promising instruments to improve communication, continuity of care, and personalized healthcare delivery in culturally diverse populations [59]. Moreover, these technologies may support longitudinal monitoring and early identification of suboptimal health conditions in vulnerable migrant groups.

### Psychosocial exposome, neuroinflammation, and cardiometabolic vulnerability

Migrants represent a particularly vulnerable population regarding psychosocial stress and mental health disorders due to cumulative exposures related to socioeconomic instability, low social status, discrimination, acculturative stress, uncertainty about the future, social isolation, and traumatic experiences associated with migration and displacement [3]. Forced migrants and refugees are especially susceptible to mental suffering, including depression, anxiety, post-traumatic stress disorder, and insomnia. In a study involving forced migrants from Sub-Saharan Africa and Southeast Asia living in refugee camps in Greece, high prevalence of depression, generalized anxiety, and sleep disturbances was observed [26]. A recent study involving female refugees from Afghanistan, Iraq, Iran, Somalia, and Eritrea residing in Germany demonstrated the profound impact of trauma-related exposures on mental health outcomes. The authors reported that the cumulative number of traumatic events was the strongest predictor of somatization, psychological distress, and reduced quality of life. Furthermore, serial mediation analyses revealed that traumatic experiences may negatively affect quality of life through interconnected pathways involving somatization and psychological distress, suggesting that migration-related adversity may become progressively embedded through physiological processes over time [60]. Similarly, among humanitarian migrants in the Orientale region, anxiety and depression were strongly associated with refugee status, overcrowded housing conditions, psychosocial stress, and low income [61]. Studies focusing on the Venezuelan diaspora have also emphasized the relevance of discrimination, hostile reception, and social exclusion as important determinants of mental health deterioration in migrant populations [62].

In this context, mental health has been an important subject of study as a risk factor for CVD. At the same time, a previous diagnosis of CVD is more common in individuals with depression, bipolar disorder, or schizophrenia [63]. Depression, anxiety, and stress are factors that can lead to increased CVD risk through several metabolic pathways [63, 64]. The Research on Obesity and Diabetes among African Migrants (RODAM) study, with 2,315 migrants and 1,549 non-migrants, found that migrants with a greater perception of discrimination have an increased risk of CVD. Racial prejudice may have more detrimental effects on the cardiovascular health of migrants when compared to non-migrants, most likely due to the inability to find strategies to cope with such prejudice and due to increased psychosocial stress [65].

In general, migrants live in unhealthy and unsafe environments characterized by neighborhoods with lower income and/or high crime. Recently, this issue was investigated, highlighting that individuals living in an unfavorable environment showed increased amygdala activation assessed by PET/CT, and this event was related to increased arterial inflammation and CVD events [66]. Additionally, a biological pathway linking these exposures to cardiometabolic vulnerability involves chronic activation of both the hypothalamic–pituitary–adrenal (HPA) axis and the sympathetic-adreno-medullary (SAM) system. Persistent exposure to psychosocial stressors may stimulate hypothalamic release of corticotropin-releasing hormone (CRH), followed by adrenocorticotropic hormone (ACTH) secretion and sustained cortisol production, while simultaneously increasing sympathetic activity and catecholamine release. These neuroendocrine responses contribute to increased peripheral vascular resistance, elevated blood pressure, endothelial dysfunction, impaired glucose metabolism, insulin resistance, and visceral adiposity [64, 67]. In addition, chronic stress is related to increased activation of pathways related to inflammatory responses, such as gene transcription related to toll-like receptors (TLR), including nuclear factor kappa B (NF-κB) [68]. These mechanisms lead to increased release of inflammatory cytokines such as interleukin-6 (IL-6), which may contribute to beta-adrenergic signaling in monocytes and, consequently, stimulate the atherosclerosis process [68]. Such findings reinforce the concept that psychosocial stress may induce measurable neuroimmune alterations potentially relevant for predictive diagnostics and early prevention within the PPPM framework [7, 69].

Furthermore, the “gut-brain axis” may also influence psychological disorders and increased cardiovascular risk, as the brain influences intestinal function through neurotransmitters, hormones, and the autonomic nervous system within the intestine [70]. Therefore, psychological disturbances may be involved in increased intestinal permeability through the reduction of tight junction proteins of the intestinal epithelium, including claudin, which impairs intestinal motility and mucin production, both affected in the intestinal dysbiosis. Increased intestinal permeability favors the translocation of bacteria and their metabolites, such as lipopolysaccharides (LPS) and trimethylamine (TMA), into the bloodstream, thus initiating systemic inflammatory responses and atherosclerotic processes [70]. Alterations in gut permeability, microbiota composition, circulating inflammatory mediators, sleep disturbances, and stress-related neuroendocrine pathways therefore represent measurable biomarkers of psychosocial exposome burden and early cardiometabolic vulnerability in migrant populations, potentially supporting risk stratification and targeted prevention strategies within the PPPM framework [69, 71].

### Built environment, lifestyle exposome, and cardiometabolic vulnerability

The built environment encompasses the physical and social spaces in which individuals live and perform daily activities, including housing conditions, transportation systems, workplaces, educational settings, and recreational areas. Within the exposome framework, these environments represent important sources of cumulative exposures capable of influencing biological responses and cardiometabolic health across the life course. Environmental characteristics such as urbanization, walkability, access to green spaces, pollution, neighborhood safety, and food availability may directly and indirectly shape health-related behaviors and physiological processes associated with cardiovascular risk.

Lifestyle-related factors, including dietary patterns, physical activity, tobacco use, alcohol consumption, sleep quality, and sedentary behavior, mediate the interaction between environmental exposures and individual health outcomes. In migrant populations, abrupt transitions between distinct social and built environments may promote significant lifestyle modifications and exposome reconfiguration, thereby influencing cardiometabolic vulnerability.

#### Smoking and tobacco-related exposome

Tobacco use remains one of the leading preventable causes of death worldwide, responsible for approximately 8.71 million deaths in 2019, of which over 1.68 million were attributable to ischemic heart disease [72]. Both active and passive smoking promote cardiovascular injury through multiple mechanisms, including endothelial dysfunction, oxidative stress, vascular inflammation, platelet activation, and hemodynamic overload, creating a pro-atherothrombotic environment that accelerates disease progression [73]. Carbon monoxide further contributes by inducing tissue hypoxia, while nicotine exacerbates sympathetic activation and vascular damage. Importantly, evidence shows that electronic nicotine delivery systems, such as e-cigarettes, share similar addictive potential and cardiovascular risks as conventional cigarettes, refuting the notion of them being safer alternatives [74]. Recent studies also demonstrate that even moderate tobacco exposure is associated with systemic inflammation, thrombosis, and subclinical atherosclerotic injury, reinforcing the urgent need for comprehensive tobacco control strategies [75].

Migrant workers represent a particularly vulnerable population, often exposed to adverse social determinants of health. These determinants include not only traditional factors such as income, education, and gender, but also conditions associated with the migration process, including legal status, language barriers, discrimination, and limited access to healthcare services [76]. These structural and psychosocial barriers may amplify risk behaviors, including tobacco use, thereby exacerbating health inequities.

A study conducted in Qatar with 778 male migrant workers from South Asia diagnosed with ischemic stroke found that 41.3% were smokers. Notably, smokers experienced stroke onset 2.03 years earlier compared to non-smokers (95% CI = 0.60–3.46; *p* = 0.005). Multivariate analysis confirmed that current smoking was an independent predictor of earlier stroke onset (β = 2.03; SE = 0.74; *p* = 0.006), regardless of other traditional risk factors such as hypertension, diabetes, obesity, dyslipidemia, coronary artery disease, and alcohol use [77]. Similar results were observed among migrant workers in South Korea, where undocumented individuals were significantly more likely to be current smokers [78]. These data suggest that migration status and legal residency directly influence the adoption of health risk behaviors, including smoking.

Another relevant finding involves Finnish migrants living in Sweden, who exhibit higher rates of smoking and smoking-related mortality among men compared to the native Swedish population. This suggests the persistence of behavioral patterns acquired before migration, which may continue even after geographic relocation [79].

Another critical and often underestimated aspect is related to exposure to secondhand smoke. Data from the 2013 China Migrants Dynamic Monitoring Survey revealed a high prevalence of both active smoking (34.1% of the sample; 55% among men and 4% among women) and secondhand smoke exposure (65% among non-smokers). Factors such as migration type and length of stay at the new location were associated with a higher likelihood of secondhand smoke exposure, underscoring the need for more effective public policies to control tobacco use among internal migrants [80].

Despite the consolidated knowledge about the deleterious effects of smoking on the cardiovascular system, including endothelial damage, autonomic dysfunction, increased oxidative stress, and systemic inflammation, there are still significant gaps in the literature regarding the understanding of behaviors related to CVD risk among migrant populations. Integration of smoking history, psychosocial assessment, biomarkers of inflammation and oxidative stress, and digital health monitoring may support earlier identification of vulnerable migrant profiles and implementation of targeted preventive interventions. Culturally adapted smoking cessation programs and personalized prevention strategies may therefore represent important approaches to reduce long-term cardiovascular risk in migrant populations.

#### Diet, food environment, and dietary acculturation

Diet is a major determinant of cardiovascular and metabolic health, and migration frequently intensifies lifestyle transitions capable of reshaping dietary behaviors and cardiometabolic risk. Movement from traditional environments to urbanized or Westernized settings is often accompanied by progressive abandonment of culturally inherited dietary practices and increased consumption of ultra-processed foods, saturated fats, refined carbohydrates, and energy-dense diets [81]. These shifts alter the intestinal microbiota, promote dysbiosis, and foster pro-inflammatory states, contributing to obesity, metabolic syndrome, and cardiovascular disease [82, 83]. Conversely, the preservation or adoption of healthier dietary patterns, such as the Mediterranean diet, rich in fruits, vegetables, whole grains, seafood, and olive oil, has been shown to protect against CVD and improve quality of life [84].

Dietary exposures are particularly relevant because they may contribute to suboptimal health conditions and cumulative cardiometabolic vulnerability [8, 17]. Recent advances in precision nutrition demonstrate that individual responses to dietary exposures are strongly influenced by genetic background, gut microbiota composition, metabolic status, psychosocial determinants, environmental context, sleep patterns, and behavioral factors, reinforcing the limitations of “one-size-fits-all” nutritional approaches [85]. In this context, migration-related dietary transitions may exert heterogeneous biological effects across individuals and populations.

Eating habits of migrant populations can be strongly influenced by the host countries, characterizing a process of food acculturation [86]. In this sense, a literature review with migrant populations in Europe showed that the increase in energy and fat intake, to the detriment of the reduction of carbohydrates from whole grains and legumes, was the primary dietary trend observed after migration. This change in eating habits was associated with a higher risk of obesity, T2D, and CVD [87]. In addition, Osei-Kwasi et al. (2020) described that different cultural origins, host country reception, and length of stay in the country can influence the eating behavior of migrant populations [88].

In this sense, migration is frequently accompanied by a progressive transition from traditional dietary patterns to more Westernized diets, characterized by increased consumption of ultra-processed foods, saturated fats, and refined carbohydrates, and reduced intake of fiber-rich foods [89, 90]. This dietary pattern leads to a reduction in microbial fermentation capacity and decreases the production of beneficial metabolites, particularly short chain fatty acids (SCFA) such as butyrate, which play essential roles in maintaining intestinal barrier integrity, regulating immune responses, and modulating glucose and lipid metabolism [89, 90]. Consequently, dietary transitions related to migration can promote intestinal dysbiosis, increased intestinal permeability, and translocation of microbial products such as LPS and trimethylamine (TMA), thereby generating chronic low-grade inflammation with elevated interleukin 6 (IL-6) and tumor necrosis factor-alpha (TNF-α), IL-1β, and IL-18. The inflammatory process is intrinsically linked to endothelial dysfunction, insulin resistance, and ultimately, an increased cardiometabolic risk [10].

An observational study comparing Ghanaian individuals living in Accra and London revealed that those residing in the United Kingdom consumed higher amounts of energy, protein, and saturated fat, while carbohydrate intake was relatively lower. After migration, the consumption of energy, macronutrients, and dietary fiber from traditional foods declined significantly. This shift was accompanied by an increased incorporation of non-traditional foods into the diet, such as breakfast cereals, wholemeal bread, and processed meats [91].

It is important to highlight that research on dietary patterns in migrant populations should be encouraged, since this is a modifiable risk factor for CVD. Targeted nutritional interventions and access to quality food can improve the health of migrant populations and should be included in the public policies of countries [92]. In addition, it is worth noting that, to date, studies on dietary acculturation have identified significant gaps in information and measurement methods, with the vast majority relying on qualitative studies [93]. Precision nutrition approaches integrating dietary assessment, microbiota profiling, metabolomics, and behavioral data may therefore support more individualized preventive strategies for cardiometabolic diseases [85]. The development of modern and validated tools is important to encourage more robust studies that better explain the association between dietary acculturation and the prevalence of diseases in migrant populations.

In a more recent study of Hispanic and Latino populations living in the United States, it was shown that dietary acculturation patterns are associated with changes in the composition of the intestinal microbiota, with 17 species of bacteria enriched and 52 depleted, including *Clostridia* and *Prevotella*, which utilize fiber for the production of SCFA, and derived metabolites involved in fatty acid and glycerophospholipid metabolism [94]. These microbiota-related changes were associated with increased cardiovascular risk and higher dietary acculturation scores, supporting the concept that migration-related dietary exposures may interact with gut microbiota and inflammatory pathways to influence cardiometabolic vulnerability. Emerging evidence further indicates that gut microbiota responses to dietary interventions vary substantially across individuals, reinforcing the importance of personalized nutrition approaches capable of integrating acculturation-related variability into preventive cardiometabolic strategies [85].

#### Sedentary lifestyle

Studies on physical activity among migrant populations are still limited, and in general, evaluate this variable within a broader lifestyle context. However, it has been elucidated that there are real barriers that culminate in the sedentary lifestyle of the migrant population, including the lack of social connections for participation in physical activities and the poor availability of facilities for practicing sports. In addition, changes in daily life activities and transportation, such as the use of cars instead of walking to work, contribute to a sedentary lifestyle [95].

Urban planning has become an even more critical challenge in the field of public health. Characteristics of the built environment, walkability, and exposure to nature influence the prevalence and incidence of CVD [96]. In this scenario, built environments that offer green spaces and/or walkability significantly improve cardiovascular health. However, there is still significant inequality in access to green spaces and walkability, especially among vulnerable populations such as migrants. An intersectional approach is necessary in public policy to understand and act on equitable urban planning [97, 98].

From a PPPM perspective, physical inactivity represents a modifiable behavioral exposure that may contribute to suboptimal health conditions long before overt cardiometabolic disease develops. Recent evidence demonstrated that physical activity attenuates, although not completely eliminate, the increased cardiovascular risk associated with obesity and central adiposity, reinforcing the importance of integrated behavioral and metabolic phenotyping for predictive diagnostics and targeted prevention. The authors highlighted that combined assessment of physical activity patterns and adiposity measures may support early identification of high-risk phenotypes and facilitate personalized prevention strategies within the PPPM framework [99].

#### Sleep habits

Sleep quality and duration have emerged as critical determinants of cardiovascular health, comparable in importance to traditional factors such as diet and physical activity [100]. Sleep disorders, including insomnia, sleep fragmentation, obstructive sleep apnea (OSA), and circadian rhythm disruptions, are strongly associated with CVD through complex pathophysiological pathways [101]. These relationships become particularly relevant in migrant populations, where the convergence of psychosocial stressors, environmental challenges, and healthcare access barriers creates a unique exposome that may amplify cardiovascular risk through sleep-mediated mechanisms [102].

The migrant exposome, encompassing environmental, occupational, and psychosocial stressors, further modulates these physiological pathways. Shift work, common among migrant workers, disrupts circadian rhythms and exacerbates metabolic dysfunction [103]. Overcrowded housing conditions and noise pollution impair sleep quality and duration, while psychological stressors such as acculturative stress and perceived discrimination contribute to insomnia and sleep fragmentation. This reality is particularly evident among Latin American immigrants in the United States, where acculturative stress increases the risk of sleep disorders by 40% [104]. A study of Hispanic/Latino adults in the United States showed that living in neighborhoods perceived as unsafe or noisy is associated with poorer sleep quality, including shorter sleep duration and a higher prevalence of insomnia. The risk of sleeping less than six hours was 7.7% points higher among those living in unsafe areas, and the risk of insomnia was 4.4% points higher in noisy areas [105]. These factors collectively create a vicious cycle where inadequate sleep increases cardiovascular risk, which in turn may further disrupt sleep through mechanisms such as nocturia or OSA-related arousals [37].

Furthermore, a systematic review of the high prevalence of sleep problems among adult immigrants in the United States highlighted that acculturative stress is consistently associated with poorer sleep quality and continuity, increased daytime sleepiness, and sleep disturbances. Although there is a lack of consensus on the acculturation measures used, the results indicate that the process of cultural adaptation plays a relevant role in sleep health inequalities among immigrants [106]. Thus, in a sample of 27,761 immigrants in the United States, a high prevalence of sleep disorders was observed, with significant variations by ethnic-racial group and length of residence in the country. Filipino and Hispanic/Latino immigrants had, respectively, the highest prevalence of short and long sleep, while those with 15 or more years of residence reported poorer sleep quality. These findings suggest that length of exposure to the North American context may exacerbate sleep inequalities among migrant populations [107]. Another systematic review of migrants and refugees reported prevalence rates of sleep disorders ranging from 39% to 99%, highlighting sleep disturbances as one of the most common health problems in these populations. Importantly, the review concluded that sleep disturbances among refugees are more strongly associated with trauma-related mental health conditions, particularly post-traumatic stress disorder (PTSD), than with psychosocial concerns alone, with sleep disorders strongly associated with trauma exposure, psychosocial stress, metabolic disturbances, and mental health disorders [108].

In this senses, as demonstrated in studies of Chinese migrant workers, interventions specifically addressing sleep duration (< 6 h associated with 26–55% higher hypertension risk) could significantly impact cardiovascular prevention [109]. The HELIUS study, which included 12,805 migrants in the Netherlands, demonstrated that this transition interacts with short sleep (< 7 h), increasing the risk of obesity by 45% in Dutch populations and 21–25% in Surinamese populations [110].

The physiological pathways linking poor sleep to cardiovascular dysfunction are multiple and interrelated. At the neuroendocrine level, sleep deprivation and fragmentation trigger sustained activation of the sympathetic nervous system, leading to increased catecholamine release, reduced heart rate variability, and chronic vasoconstriction. This sympathetic overactivity not only contributes to hypertension but also perpetuates a state of cardiovascular hyperreactivity to stress, particularly exacerbated in migrants facing acculturative stress [111].

Inflammatory pathways represent another crucial link between sleep disturbances and cardiovascular diseases. Poor sleep quality increases the expression of inflammatory cytokines, such as IL-6 and TNF-α, while elevating C-reactive protein (CRP) levels, thereby creating a chronic, low-grade inflammatory state [111, 112]. This persistent inflammation compromises endothelial function by reducing nitric oxide bioavailability and promoting oxidative stress, thereby accelerating the progression of atherogenesis. For migrant populations, this inflammatory burden may be aggravated by psychosocial stressors such as discrimination or family separation, creating a synergistic detrimental effect on vascular health [18].

The complex interaction between migration-related stressors, physiological dysregulation, and sleep disturbance creates a unique risk profile demanding greater attention in both research and public health initiatives [109]. Addressing sleep health in migrants through culturally sensitive approaches may offer a promising pathway to reduce CVD risk disparities and improve health outcomes in these vulnerable populations [102].

In this sense, implementation of sleep-focused strategies in migrant populations should include earlier identification of vulnerable individuals through culturally adapted screening tools, sleep questionnaires, wearable monitoring technologies, and assessment of psychosocial and environmental stressors. Preventive approaches may include sleep health education, circadian hygiene interventions, workplace-based programs for shift workers, and targeted strategies addressing acculturative stress, discrimination, and housing conditions [108]. Furthermore, personalized medical approaches integrating digital health technologies, telemedicine, multilingual cognitive behavioral therapy for insomnia (CBT-I), and individualized management of obstructive sleep apnea may contribute to reducing long-term cardiometabolic risk and improving health outcomes in migrant populations [108].

### Environmental exposome, climate change, and pollution-related cardiovascular vulnerability

Environmental exposures represent major determinants of cardiovascular and metabolic health and constitute essential components of the exposome framework. Climate change, urbanization, environmental pollution, and ecological degradation are increasingly recognized as interconnected drivers of migration, social vulnerability, and chronic disease burden. Within the framework of PPPM/3PM, these exposures are particularly relevant because they may contribute to cumulative biological vulnerability and suboptimal health conditions [5, 15].

Climate change has emerged as an important driver of migratory movements worldwide. Probabilistic models estimate that by 2050 more than 250 million individuals may become “environmental refugees” due to rising temperatures, droughts, floods, food insecurity, and extreme weather events that compromise living conditions, economic stability, and health systems [113]. Balsari et al. (2020) suggested that climate change is expected to lead to the migration of populations from rural to urban areas, which will eventually become unsustainable urban centers, which could further influence migration in search of other locations. The authors also emphasize that it is essential to acknowledge that climate change exacerbates the need for or decision to migrate and disproportionately affects more vulnerable communities [114].

A recent study examining the migration from Latin America to the Atlanta metropolitan area revealed vulnerabilities related to climate change that influence migration decisions, particularly in relation to social determinants of health and well-being [114]. Similar findings were observed among migrant populations from deltaic regions in Bangladesh and Ghanain to others countries (unspecified). Although individuals did not identify climate change as the primary trigger for displacement, adverse climate-related events negatively affect economic security, food security, and agricultural and livestock production [115].

Another study showed that migrants from the Middle East and North Africa, one of the regions most affected globally by climate change and wars, reported that climatic events such as catastrophic floods, extreme heat, cold temperatures, and dust storms were identified as major drivers of displacement and interruption of healthcare services [116]. In this population, all individuals had high blood pressure, which was attributed to the experience of war and violence, as well as poor environmental conditions, including poor air quality and heat during the migratory journey [116]. Additionally, a study of Nepalese migrant workers demonstrated that the average annual mortality rate for this population in Qatar was 150 deaths/100,000, with the leading cause being CVD, and correlated with average monthly heat levels. Furthermore, it was suggested that these deaths could be prevented with effective heat protection measures, as climate change leads to increased health risks [117].

The association between the development of CVD and climate change in recent decades has been a topic of current and essential interest [118–120]. Extreme heat exposure elicits physiological responses characterized by peripheral vasodilation, resulting in an increase in blood flow [119].

In addition, there is a decrease in ventricular preload and systemic vascular resistance due to the increase in peripheral circulation, thus requiring a greater cardiac output to maintain homeostasis of blood perfusion to the organs by increasing the heart rate [118]. This hyperdynamic state can lead to hemoconcentration due to hyperthermia and sweating. The state of hemoconcentration ends up contributing to hypercoagulation, which is involved in the increased risk of thrombosis, acute myocardial infarction, and ischemic stroke [118, 119]. Furthermore, an inflammatory response may be activated by extreme heat, increasing levels of cytokines such as IL-6, TNF-α, and IL-1β, which, together with the systemic response, may increase ischemic morbidity and mortality during heat waves [121].

In this context, pollution represents a single or combined inadequate sources of air, water, food, and cosmetic and cleaning products that can induce accumulation and synergistic effects on health [32]. Environmental pollution poses a significant threat to cardiovascular health, especially among migrant populations, who tend to be more exposed to air pollutants, heavy metals, and pesticides. Air pollution, which comes from the burning of fossil fuels in industrial activities, traffic, and power generation, is composed of gases and particles such as Particulate Matter (PM) 2.5, nitrogen dioxide (NO2), nitrogen monoxide (NO), ozone (O3), and sulfur dioxide (SO2) [122].

Studies show that poor air quality can drive population migration [123] and is associated with an increased risk of diseases such as stroke and coronary heart disease (CHD) [122]. Migrants in more polluted areas, such as China, demonstrate a higher risk of health problems [124], highlighting the importance of clean air as a factor in attracting or repelling migrants.

Frequent exposure to air pollution induces a chronic inflammatory state, which is associated with the progression or vulnerability of atherosclerotic plaques, thereby promoting acute coronary syndrome events [9]. Inhaled air pollutants induce oxidative stress through the production of reactive oxygen species (ROS), which can contribute to the activation of inflammatory pathways, with increased stimulus to the synthesis of IL-6, IL-1, TNF-α, interferon gamma, and granulocyte-macrophage colony-stimulating factor [125]. These events stimulate the release of vasoactive substances, such as endothelin and C-reactive protein, into the bloodstream. ROS also induces toll-like receptor 2 and 4 (TLR2/4) and transient receptor potential (TRP), related to the activation of the inflammatory pathway. These, result in the release of adhesion molecules, induction of leukocytes and platelets, a systemic pathway related to blood coagulation, atherosclerosis, arrhythmias, myocardial infarction, and endothelial dysfunction [125].

Furthermore, exposure to heavy metals such as mercury, lead, cadmium, and arsenic from various environmental sources has been linked to an increased risk of CVD. Migrant populations have higher plasma concentrations of these metals, attributed to factors such as dietary habits, housing, and work conditions [126, 127]. Refugee children or children from racial minorities in the USA, for example, have a higher prevalence of lead in their blood, with profound health implications [128]. These inequalities reinforce the need for specific public policies to mitigate exposure in vulnerable groups.

Exposure to heavy metals can lead to the induction of gluconeogenesis and cellular dysfunction, resulting in the inhibition of beta oxidation through the suppression of peroxisome proliferator-activated receptor gamma (PPAR gamma) expression [129]. Furthermore, some specific heavy metals, such as metalloestrogens, can increase cardiovascular risk through endocrine disruption [130]. In addition, these toxic metals increase the production of ROS and lipid peroxidation, which is associated with the atherosclerotic process. Heavy metals are associated with damage to vascular tissues, resulting in endothelial dysfunction and the degradation of proteins and nucleic acids [131]. Furthermore, the deficiency of essential metals concomitant with the excess of toxic metals is involved in the impairment of the immune system and accumulation of immune complexes, including IL-6, TNF-α, monocyte chemotactic protein 1 (MCP-1), vascular cell adhesion molecule 1 (VCAM-1), and intercellular adhesion molecule (ICAM), which are related to increased CVD risk [131].

It has recently been demonstrated that among the detrimental health impacts associated with heavy metal exposure is a decrease in the antioxidant defense. Heavy metals can interact with antioxidant enzymes, including intracellular glutathione (GSH), superoxide dismutase (SOD), catalase, glutathione peroxidase (GPx), and glutathione reductase, inhibiting their function. Furthermore, another critical point is heavy metal toxicity, which involves the replacement of essential metals by structurally similar heavy metals at protein binding sites, causing damage to lipids, proteins, and DNA [130].

Additionally, pesticides such as organophosphates, pyrethroids, and organochlorines are widely used in agriculture, accumulating in the environment and in food. Migrants, especially agricultural workers and their children, are more exposed to these compounds, which have been associated with genetic damage, metabolic alterations, and increased risk of CVD and metabolic diseases [132, 133].

Concerning human health, pesticide toxicity can generate acute and chronic diseases, including CVD, such as heart failure [134]. Pesticides can act by inhibiting hormone activity, leading to changes in immune function, increased oxidative stress, inflammation, and genetic disruption [134]. In a systematic review, free radicals from pesticides played an important role in human health, including the development of myocardial infarction, congestive heart failure, stroke, arrhythmia, and sudden death [135]. In an observational study, exposure to pesticides increases the risk of CVD through the induction of multiple signaling pathways and gene expression related to mitogen-activated protein kinase (MAPK), AMP-activated protein kinase (AMPK), forkhead box O (FoxO) signaling, apoptosis, and phosphoinositide 3-kinase/protein kinase B (PI3K-Akt) [136].

Moreover, the development of plastic has transformed numerous processes globally involving the environment and individuals. Although its presence represents a symbol of modern societies, its impact on health, environment, and sustainability has contributed to rethinking its production and use at the global level. Plastic is present in food packaging and construction, with its distribution worldwide varying significantly according to the economic development level of countries and the transition from urban to rural areas. Chemicals such as bisphenol A and phthalates promote a range of negative effects on health outcomes through their endocrine-disrupting properties; however, current investigations about micro- and nano plastics (MNPs) identified a potential deleterious role of these components for cardiovascular health and their CVD risk factors [137].

In fact, increased evidence confirms the detrimental role of MNPs for the cardiovascular system. Short-term oral exposure to polystyrene in mice and rats resulted in deposition in the blood, heart, and isolated cardiomyocytes [138]. Furthermore, MNPs promote oxidative stress in multiple cell types, including endothelial cells [139], and chronic and low-grade inflammatory response, that induce cellular senescence [140]. Many previous studies confirmed that the use of different types of MNPs exerts a negative role on endothelial cells, favoring their dysfunction, a key phenomenon for the atherosclerotic process [141].

Although the literature does not establish a clear link between migratory movements and exposure to MNPs, previous studies suggest that the increased CVD risk observed in migrants can be, at least in part, explained by the accumulation of MNPs in humans. At this moment, the current evidence does not permit establishing a hard endpoint connecting MNPs and CVD risk in immigrants. However, recent estimates suggest that 86 to 710 trillion MNP particles contaminate European agriculture, impacting the general population [142].

In view of this, integration of environmental assessment with geospatial analysis, exposome profiling, biomarkers of oxidative stress and inflammation, wearable sensors, digital health technologies, and multi-omics approaches may support predictive diagnostics, risk stratification, and earlier identification of vulnerable migrant profiles. Furthermore, climate-adapted public health strategies, environmental protection policies, occupational interventions, pollution monitoring, and personalized preventive approaches may contribute to reducing environmental health inequalities and long-term cardiometabolic risk in migrant populations.

## Conclusions and expert recommendations within the PPPM framework

This review highlights migration as a dynamic process of exposome reconfiguration capable of reshaping cardiometabolic vulnerability through cumulative environmental, psychosocial, behavioral, occupational, and biological exposures across the life course. Rather than resulting from isolated behavioral or genetic determinants, cardiovascular risk in migrant populations emerges from complex interactions involving social inequalities, environmental pollution, psychosocial stress, dietary acculturation, sleep disruption, occupational burden, and barriers to healthcare access. Importantly, cardiometabolic vulnerability is influenced not only by conditions in the host country, but also by environmental, socioeconomic, and psychosocial factors experienced before and during migration. Forced migrants and refugees may be particularly vulnerable due to traumatic experiences, unstable living conditions, and increased psychosocial stress burden.

**Fig. 3:**
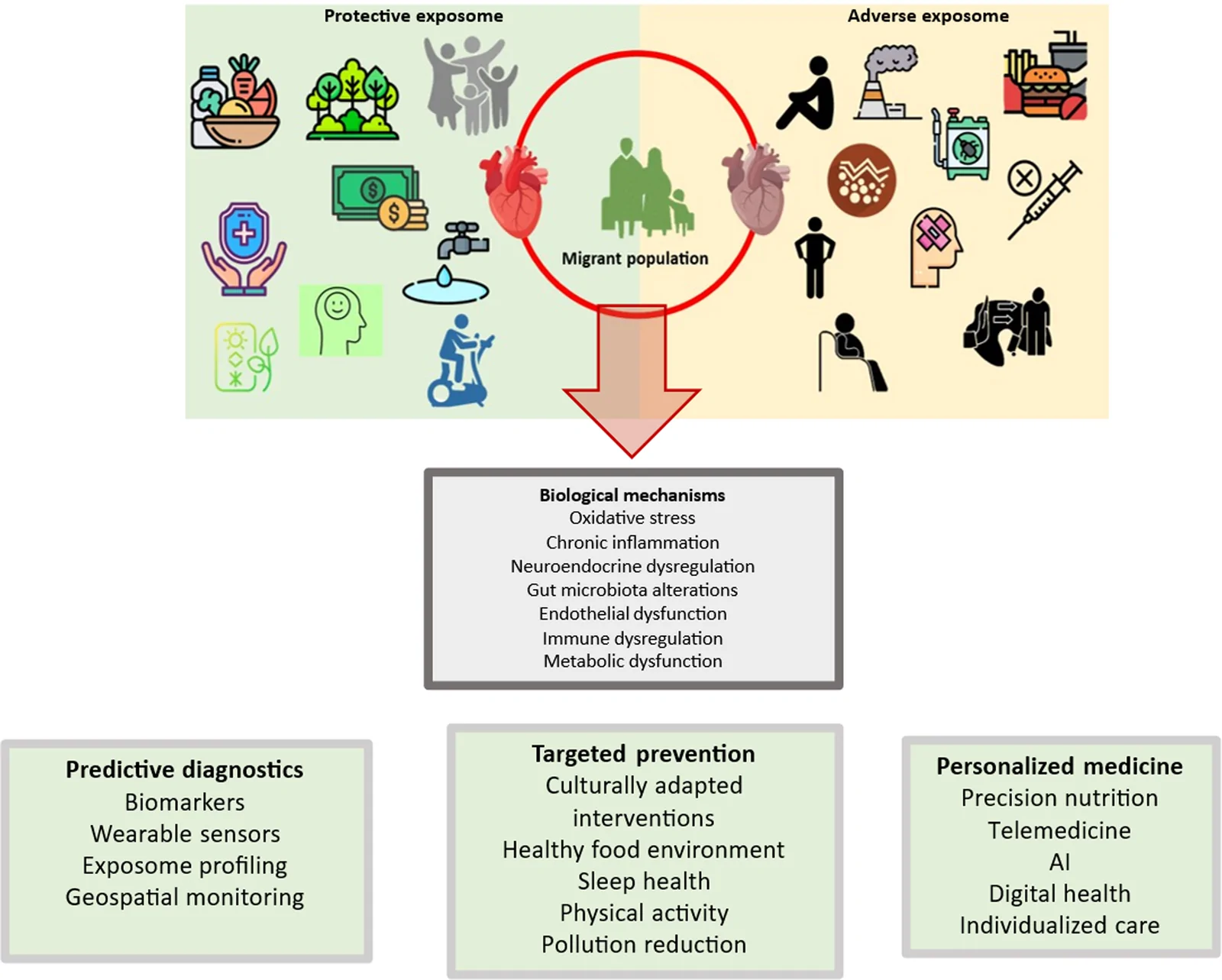
Migration-related exposome and its implications for cardiometabolic vulnerability within the framework of predictive, preventive and personalized medicine (PPPM/3PM). Migration dynamically reshapes environmental, psychosocial, behavioral, occupational, and sociocultural exposures across the life course. Protective and adverse exposome factors interact with biological mechanisms including oxidative stress, chronic inflammation, neuroendocrine dysregulation, gut microbiota alterations, endothelial dysfunction, metabolic dysfunction, and suboptimal health conditions, thereby influencing cardiometabolic risk in migrant populations. Integration of biomarkers, multi-omics approaches, wearable sensors, geospatial monitoring, artificial intelligence, and digital health technologies may support predictive diagnostics, targeted prevention, and personalized healthcare strategies aimed at reducing long-term cardiovascular vulnerability

Environmental pollutants, unhealthy food environments, sedentary behavior, inadequate sleep habits, occupational exposures, and limited healthcare access may amplify chronic inflammation, oxidative stress, metabolic dysfunction, neuroendocrine dysregulation, and endothelial injury, thereby increasing long-term cardiovascular risk. Integration of exposome science with PPPM/3PM may therefore provide novel opportunities for earlier identification of vulnerable migrant profiles, targeted prevention, and development of culturally adapted personalized healthcare strategies before clinically manifest cardiovascular disease develops (Fig. 3).

### Predictive medical approaches

Predictive medical approaches should include early identification of migration-related exposure profiles associated with increased cardiometabolic vulnerability. Structured assessment of psychosocial stress, dietary acculturation, sleep disruption, environmental pollution exposure, occupational burden, social deprivation, and lifestyle-related factors may support earlier detection of suboptimal health conditions before overt cardiovascular disease develops. Integration of exposome assessment with inflammatory biomarkers, oxidative stress markers, metabolic profiling, gut microbiota analysis, neuroendocrine markers, wearable technologies, geospatial analysis, and digital health monitoring may further improve predictive diagnostics and risk stratification in migrant populations.

The exposome requires innovative and integrative methodologies capable of capturing dynamic environmental, behavioral, psychosocial, and biological exposures across the life course. In this context, digital technologies such as GPS-enabled smartphones, wearable sensors, mobile health applications, and telemonitoring platforms may support real-time assessment of air pollution, noise exposure, sleep patterns, physical activity, geospatial mobility, and psychosocial stressors [143, 144]. Integration of these technologies with validated questionnaires, biomarkers of exposure and effect, and multi-omics approaches may improve individualized exposure assessment and facilitate earlier identification of vulnerable migrant profiles before clinically manifest cardiovascular disease develops.

Importantly, predictive strategies should be adapted according to subgroup-specific exposome profiles. Refugees and asylum seekers may particularly benefit from assessment of trauma-related vulnerability, psychosocial stress, sleep disturbances, and inflammatory biomarkers, whereas labor migrants may require greater emphasis on occupational exposures, circadian disruption, environmental pollutants, and cardiovascular risk monitoring. For undocumented migrants, predictive approaches should also consider barriers to healthcare access and delayed diagnosis.

#### Targeted prevention

Targeted prevention strategies should prioritize reduction of modifiable environmental and behavioral risk factors disproportionately affecting migrant populations. Culturally adapted interventions promoting healthy dietary patterns, physical activity, sleep health, smoking cessation, stress reduction, and improved access to healthcare services may reduce cumulative cardiometabolic burden. Preventive programs should also consider occupational exposures, unsafe housing conditions, environmental pollution, circadian disruption, and barriers related to language, discrimination, and legal status.

Public health policies aimed at improving walkability, access to green spaces, healthy food environments, pollution control, occupational protection, housing conditions, and social integration may contribute to reducing health inequities and improving cardiovascular outcomes in migrant populations. Community-based interventions and migrant-sensitive healthcare programs should also be encouraged to improve adherence, health literacy, and long-term prevention strategies.

In addition, preventive priorities may also differ across migrant subgroups, for example, interventions for refugees and forced migrants should integrate psychosocial support, trauma-informed care, and social protection measures. Occupational health surveillance and workplace-based interventions may be particularly relevant for labor migrants, while community-based outreach programs, multilingual communication strategies, and accessible screening initiatives may be especially important for undocumented migrants. International students may benefit from interventions focused on mental health, sleep health, dietary adaptation, and social integration.

#### Personalization of medical services

Personalized medical services for migrant populations should incorporate culturally sensitive and individualized healthcare approaches capable of addressing heterogeneous exposome profiles and sociocultural contexts, account for the specific migration history, legal status, social context, and dominant exposome characteristics of different migrant subgroups. Such stratification may improve adherence, facilitate earlier identification of vulnerability, and enhance the effectiveness of personalized preventive interventions.

Particularly in migrant populations facing language and health-literacy barriers, telemedicine services, multilingual mobile applications, visual interfaces, wearable devices, remote monitoring systems, and culturally adapted health platforms may facilitate more equitable access to cardiovascular prevention and care. These tools may support cardiovascular risk assessment, lifestyle monitoring, medication adherence, and patient-provider communication while reducing dependence on advanced language proficiency [145].

Artificial intelligence and machine learning approaches may further support integration of multidimensional datasets, including environmental exposures, dietary patterns, social interactions, behavioral data, and biological markers, enabling more precise characterization of exposome-related cardiometabolic vulnerability [146]. Such technologies may facilitate personalized prevention strategies, dynamic risk stratification, and individualized healthcare approaches adapted to the heterogeneous exposome profiles and sociocultural contexts of migrant populations.

Integration of multi-omics approaches, microbiota profiling, environmental exposure assessment, and precision nutrition strategies may further support personalized cardiometabolic prevention and healthcare delivery in migrant populations. Importantly, personalized approaches should also consider migration history, psychosocial vulnerability, occupational conditions, sleep patterns, cultural health beliefs, and social determinants of health to improve adherence and long-term outcomes.

## Data Availability

No datasets were generated or analysed during the current study.
